# Supplementary material for: Seminoma and Embryonal Carcinoma Footprints Identified by Analysis of Integrated Genome-Wide Epigenetic and Expression Profiles of Germ Cell Cancer Cell Lines
Source: PLoS One. 2014 Jun 2;9(6):e98330. doi: 10.1371/journal.pone.0098330 (PMC4041891; doi:10.1371/journal.pone.0098330)
Supplement: File S1 — ZIP file containing DMRforPairs output for significant regions. Please start from the html files. (ZIP) [file pone.0098330.s008.zip › figures/42087.pdf]

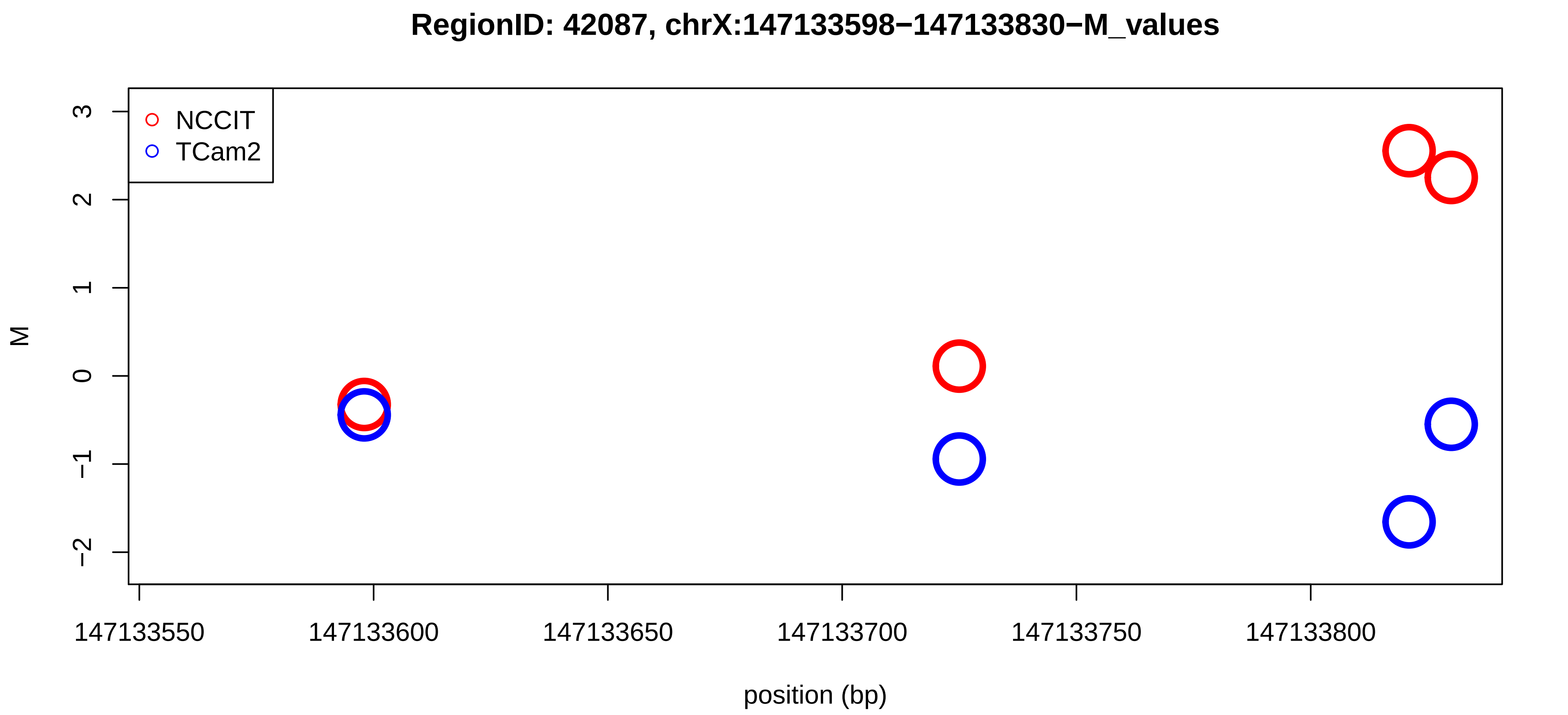

RegionID: 42087, chrX:147133598−147133830−M_values
NCCIT
TCam2
M
3
2
1
0
−1
−2
147133550
147133600
147133650
147133700
147133750
147133800
position (bp)

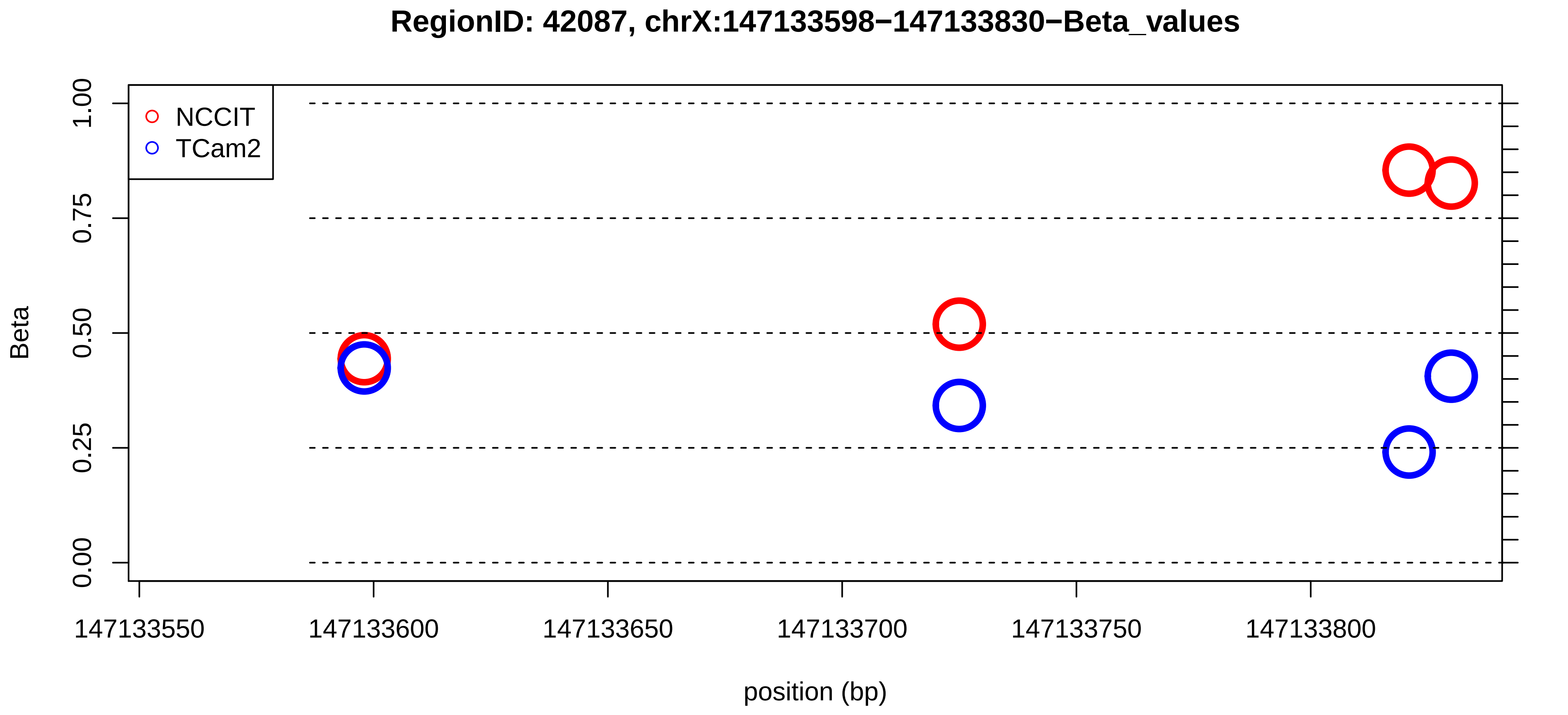

RegionID: 42087, chrX:147133598−147133830−Beta_values
NCCIT
TCam2
Beta
1.00
0.75
0.50
0.25
0.00
147133550
147133600
147133650
147133700
147133750
147133800
position (bp)
